# High affinity associations with α-SNAP enable calcium entry *via* Orai1 channels

**DOI:** 10.1371/journal.pone.0258670

**Published:** 2021-10-15

**Authors:** Ramanagouda Ramanagoudr-Bhojappa, Yong Miao, Monika Vig

**Affiliations:** 1 Cancer Genetics and Comparative Genomics Branch, NHGRI, Bethesda, MD, United States of America; 2 GEiC, Washington University in St. Louis, School of Medicine, St. Louis, MO, United States of America; 3 Tata Institute of Fundamental Research, Hyderabad, India; Russian Academy of Medical Sciences, RUSSIAN FEDERATION

## Abstract

Molecular steps that activate store-operated calcium entry (SOCE) *via* Orai channel supramolecular complex remain incompletely defined. We have earlier shown that α-SNAP regulates the on-site functional assembly and calcium selectivity of Orai1 channels. Here we investigate the molecular basis of its association with Orai, Stim and find that the affinity of α-SNAP for Orai and Stim is substantially higher than previously reported affinities between Stim and Orai sub-domains. α-SNAP binds the coiled-coil 3 (CC3) sub-domain of Stim1. Mutations of Tryptophan 430 in Stim1-CC3 disrupted α-SNAP association and SOCE, demonstrating a novel α-SNAP dependent function for this crucial subdomain. Further, α-SNAP binds the hinge region near the C-terminus of Orai1 and an additional broad region near the N-terminus and Valine 262 and Leucine 74 were necessary for these respective interactions, but not Orai, Stim co-clustering. Thus, high affinity interactions with α-SNAP are necessary for imparting functionality to Stim, Orai clusters and induction of SOCE.

## Introduction

The genome-wide RNAi screens initially identified Stim and Orai as two crucial components of the store-operated CRAC channel complex [1–6] and the past decade has seen rapid growth in the structure-function analysis of these two proteins. The depletion of calcium stores induces oligomerization and translocation of Stim1 to the peripheral/ junctional endoplasmic reticulum (ER) tubules localized in close proximity with plasma membrane (PM) [7]. The translocation of Stim1 to ER-PM junctions has been extensively studied, and may be facilitated by several other proteins [8]. However, the steps that follow Stim1 translocation remain far from clear.

Stim1 oligomerization remains equated with the functional exposure and binding of the Stim Orai activation region (SOAR)/ CRAC activation domain (CAD) of Stim1 with Orai1 [9, 10]. SOAR/ CAD is a ~100 amino-acid long minimal domain of Stim1 necessary for activating SOCE [11, 12] and previous reports have assigned multiple different roles to it. It is thought to be responsible for Stim1 oligomerization, entrapment of freely diffusing Orai1 in PM [13, 14], *via* direct interactions with the C-terminus [15], and induce allosteric shifts in Orai1 for activating SOCE. Yet mechanistically, how Stim1 single-handedly performs all these roles remains unclear. Further, reduced SOCE in several Stim1 and Orai1 mutants remains correlated with defects in Stim1 intrinsic intramolecular unlocking necessary to expose SOAR/ CAD or failure to bind and induce allosteric shifts in Orai1 [10], irrespective of whether the mutations directly reside within the Stim1 intra-locking or Stim, Orai interacting interfaces identified from structural studies [16, 17]. One reason for these gaps in understanding could be the extrapolation of the qualitative observations that soluble Stim1 C-terminus can induce calcium extrusion *via* Orai1 containing vesicles [11, 12, 18] to physiological activation of calcium-selective Orai1 channels *in vivo*. It is therefore not surprising that several mutants of full length Stim1 and Orai1 are now being identified [12, 19], including the three identified in this paper, that are able to co-cluster in ER-PM junctions, but fail to activate SOCE to the extent required to elicit normal physiological responses.

A few years ago, we identified α-SNAP as an essential regulator of SOCE *via* Orai1 channels [20]. In α-SNAP depleted cells, Orai1 entrapment by Stim1 is neither sufficient to retard its diffusion nor trigger calcium selective SOCE [14, 20, 21]. We further showed that unstable contacts between Stim1 and Orai1 N-terminus could, in part, underlie these defects in α-SNAP depleted cells [14]. It is therefore unlikely that Stim, Orai contacts that entrap Orai1 in ER-PM junctions are by themselves capable of inducing the hypothetical allosteric shifts required for SOCE *via* Orai1 [9, 10]. Based on these findings, we reasoned that the steps that entrap Orai1 in ER-PM junctions can be dissociated from the step of calcium selective SOCE *via* disparate molecular interactions.

α-SNAP is the only molecular component, identified thus far, that consistently co-clusters with Stim and Orai post store-depletion [14, 20]. Therefore, we sought to identify the molecular determinants of functional interaction between α-SNAP, Orai1 and Stim1. Here we identify the minimal sub-domains of Orai1 and Stim1 that bind α-SNAP and find that the affinities of these associations are significantly higher when compared to previously reported affinity between Stim1 and Orai1 sub-domains. We demonstrate that specific hydrophobic residues are involved in the functional association of Orai1, Stim1 and α-SNAP. Collectively, these findings systematically define the molecular basis of α-SNAP dependent functional assembly of Orai1 channel supramolecular complex and ascribe additional functions to the domains and residues previously found to be essential for SOCE.

## Materials and methods

### Cell lines, plasmids and transfection

Stable *Orai1-/-* and *Stim1-/-* MEF cell lines were established as described previously [14, 22]. The mEOS3.2 Orai1, Orai1-CFP, Orai-Myc-His and YFP-Stim1 plasmids have been described previously [14, 20]. The point mutants of Stim1 and Orai1 were generated using the site directed mutagenesis kit (Qiagen). For imaging, MEFs were plated on poly-D-lysine coated glass bottom dishes, transiently transfected with the desired plasmids and their mutants using Viafect transfection reagent (Promega Corp., WI) and analyzed 12–16 hour later. 1μM thapsigargin (TG) was used for calcium store depletion while imaging store-depleted cells as described before [20].

### Cloning, expression and purification of proteins

Untagged fragments of Stim and Orai, Stim1 336–485, Orai1 1–103 and Orai1 245–292 were amplified from human YFP-Stim1 and Orai1-Myc-His plasmids respectively [2, 23] and sub-cloned in pSUMO vector (Clontech). The fusion proteins were expressed in LEMO21(DE3) cells (New England Biolabs) and induced with IPTG (0.3 mM) for 14 hours at 18°C. Cells were lysed in a buffer containing 50 mM sodium phosphate buffer pH 7.4, 500 mM NaCl, 3 mM β-ME, 20 mM imidazole, 10% glycerol, Protease Inhibitor Cocktail, DNaseI and 1 mg/ml lysozyme. Lysates were centrifuged at 25,000 xg for 30 min, and supernatants were applied on to HisTrap HP column (GE Healthcare) using AKTA chromatography system (Amersham Pharmacia). The column was washed and the fusion protein was eluted using 500 mM imidazole. Purified fusion proteins were digested with Ulp1 protease to separate SUMO-tag from the desired protein. The cleaved sample was reapplied to fresh HisTrap HP column to trap SUMO-tag and Ulp1 protease. The desired protein in the flow-through fraction was collected, pooled and further purified using ion exchange chromatography.

Human α-SNAP was amplified from HEK 293 cDNA and cloned into pET28b vector with a Avitag and a C-terminal 6xHis tag. The protein was expressed as described above, and purified as reported previously [24]. The purified protein was *in vitro* biotinylated using BirA ligase and the unlabeled biotin was removed using Zeba spin desalting column according to manufacturer instructions (Thermo Fisher Scientific).

### Circular dichroism (CD) spectroscopy

CD analyses were performed using a Jasco J-810 spectropolarimeter (Jasco, Easton, MD). Far-UV CD spectra were acquired in a 1-mm-path-length cuvette, with a 1-nm bandwidth, and a scan rate of 50 nm/min. All measurements were conducted at room temperature in a buffer containing 10 mM NaH_2_PO_4_ (pH 7.4), 150 mM NaF at indicated protein concentrations. In some cases, 20% trifluoroethanol (TFE) was added before obtaining spectra. Spectra of each sample, representing the average of three scans, were baseline corrected by subtracting the spectra of buffer alone. DICHROWEB, an online server for protein structure analysis from CD spectroscopic data [25] was utilized to assess the secondary structure of the proteins from their spectra.

### Bio-Layer interferometry (BLI)

All experiments were run on Octet RED96 biosensor system (ForteBIO) at 25°C. For each binding assay, 0.25 μM of biotinylated α-SNAP (termed as ligand in the assay description) was diluted in assay buffer (PBS buffer supplemented with 2 mg/ml BSA and 0.02% Tween-20), and incubated with streptavidin biosensor tips for 3 min. After coupling, the unbound streptavidin sites were quenched with biocytin (10 μg/ml) for 3 min. The kinetic assay was performed by taking pre-association baseline in assay buffer for 60 seconds followed by association with indicated analyte (purified Orai1 or Stim1 fragments) at varying concentrations for 5 min, and dissociation in assay buffer for 10 min. Double referencing with both a reference sample and reference biosensors was performed. The reference sample was run using ligand-loaded biosensor in assay buffer without analyte. Reference biosensors were loaded with biocytin alone and run through the assay with various dilutions of analyte, matching with the ligand-loaded biosensors. The reference biosensor was used to subtract non-specific binding of analytes to the biosensors.

#### BLI assay data analysis

The association and dissociation analyses were performed using ForteBIO data analysis software v8.2. The software uses the following equation to fit the association curve to obtain *k*_obs_ value:

$$Y=Y_{0}+A(1-e^{-k_{obs*t}})$$ (1)

where, Y = level of binding, Y_0_ = binding at start of association, A is an asymptote and t = time, *k*_obs_ is the observed rate constant.

Similarly, the following equation used to fit the dissociation curve to obtain *k*_d_ value:

$$Y=Y_{0}+{Ae}^{-k_{d}*t}$$ (2)

where, Y_0_ is binding at start of dissociation, and *k*_d_ is the dissociation rate constant. The association rate constant *k*_a_ can then be calculated with the following equation:

$$k_{a}=\frac{k_{obs}-k_{d}}{\left[Analyte\right]}$$ (3)

The equilibrium dissociation constant *K*_D_ can be calculated using *k*_a_ and *k*_d_:

$$K_{D}=\frac{\left[A]*[B\right]}{\left[AB\right]}=\frac{k_{d}}{k_{a}}$$ (4)

Experimental data were first fit to a model for a single ligand binding to a single receptor (1:1 binding model) to obtain an equilibrium dissociation constant value. Subsequently, data were fit to the simplest two-site model of ligand binding. The goodness-of-fits of these two models were analyzed visually by comparison with experimental data and statistically by R-squared value. The simpler 1:1 binding model was chosen as the best fit unless the 2:1 binding model fit significantly better.

### Pull-down assay

The fragments of Stim1 (342–448, 344–382 and 408–442) and Orai1 (1–103, 1–47, 48–103, 228–301, 245–272 and 272–292) were amplified from full length constructs and cloned into pMAL-c5X (New England Biolabs) or pGEX-4T2 (Addgene) vector, in-frame with MBP or GST protein coding sequences, respectively. The fusion proteins were expressed in LEMO21(DE3) cells (New England Biolabs) by inducing with IPTG (0.3 mM) for 14 hours at 18°C. The MBP- or GST-tagged proteins were purified by immobilizing on amylose (NEB) or glutathione resin (Qiagen), respectively. The resin bound proteins were incubated with 20–50 nM of α-SNAP protein in Ringer’s buffer containing 0.1% NP-40 and 2 mM imidazole for 1 hr at 4°C. The resin was washed thrice with Ringer’s buffer, boiled in SDS sample buffer and subjected to SDS-PAGE followed by Western Blot analysis.

### Measurement of single cell SOCE and [Ca^2+^]_i_

Cells were loaded with 1μM Fura-2-AM (Life Technologies) in Ringer’s buffer (135 mM NaCl, 5 mM KCl, 1 mM CaCl2, 1 mM MgCl2, 5.6 mM Glucose, and 10 mM Hepes, pH 7.4) for 40 min in the dark, washed, and used for imaging. Baseline images were acquired for 1 minute and then cells were simulated with 1 micromolar TG and imaged simultaneously in nominally calcium free Ringer’s buffer for 5 to 6 minutes. Subsequently, extracellular calcium was replenished and cells were imaged for an additional 5–6 minutes. 20–30 cells were analyzed per group in each experiment. An Olympus IX-71 inverted microscope equipped with a Lamda-LS illuminator (Sutter Instrument, Novato, CA), Fura-2 (340/380) filter set (Chroma, Bellows Falls, VT), a 10X 0.3NA objective lens (Olympus, UPLFLN, Japan), and a Photometrics Coolsnap HQ2 CCD camera was used to capture images at a frequency of ~1 image pair every 1.2 seconds. Data were acquired and analyzed using MetaFluor (Molecular Devices, Sunnyvale, CA), Microsoft Excel, and Origin softwares. To calculate [Ca^2+^]_i_, Fura-2 Calcium Imaging Calibration Kit (Life technologies) was used according to manufacturer’s instructions. Briefly, standard samples containing dilutions of free Ca^2+^ (0 to 39 μM) were imaged as described above to obtain the constant *K*_*d*_. [Ca^2+^]_i_ was then determined using the following equation:

$$[{Ca}^{2+}]=K_{d}*\frac{\left[R-R_{min}\right]}{\left[R_{max}-R\right]}*\frac{F_{max}^{380}}{F_{min}^{380}}$$ (5)

where R is the ratio of 510 nm emission intensity with excitation at 340 nm versus 380 nm; R_min_ is the ratio at zero free Ca^2+^; R_max_ is the ratio at saturating free Ca^2+^; F^380^_max_ is the fluorescence intensity with excitation at 380 nm, for zero free Ca^2+^; and F^380^_min_ is the fluorescence intensity at saturating free Ca^2+^. SOCE was calculated as (SOCE = highest [Ca^2+^]_i_−basal [Ca^2+^]_i_), where highest [Ca^2+^]_i_ was the highest value after replenishing extracellular calcium and basal [Ca^2+^]_i_ was the lowest [Ca^2+^]_i_, following store-depletion in calcium free buffer.

### TIRF microscopy

Mutants of full length YFP-Stim1, mEOS3.2 Orai1 and Orai1-CFP were expressed in HEK 293 cells and imaged under resting and store-depleted conditions on a custom built TIRF microscopy setup as described previously [20]. At least 6 to 7 cells per group were imaged under resting as well as store-depleted conditions.

### Statistical analysis

Statistical significance represented as *p* value was calculated using unpaired student’s *t*-test. * *p*<0.05, ** *p*<0.01, *** *p*<0.001.

## Results

### α-SNAP binds to the CC3 domain of Stim1

**Fig 1:**
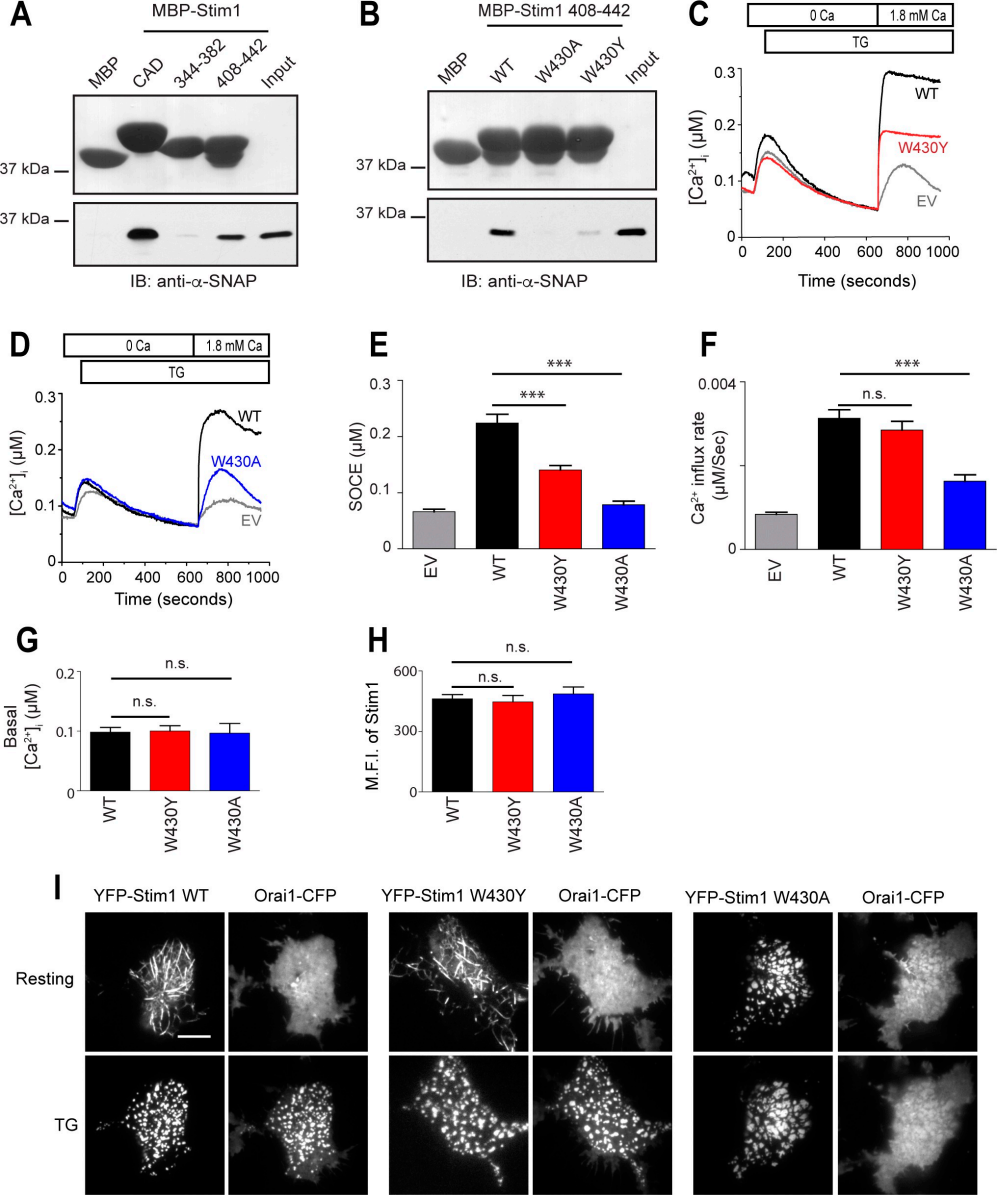
α-SNAP binds to the CC3 domain of Stim1. **(A)** *In vitro* binding of α-SNAP to MBP-tagged CAD and its sub-domains Stim1 344–382 and Stim1 408–442. (Top) Ponceau S staining showing the input of MBP-tagged fragments. (Bottom) Immuno Blot (IB) for α-SNAP showing α-SNAP protein pull down by MBP-tagged fragments. (n = 3) **(B)** *In vitro* binding of α-SNAP to MBP-tagged Stim1 408–442 and its mutants. (Top) Ponceau S staining showing the input of MBP-tagged proteins. (Bottom) IB for α-SNAP showing α-SNAP pull down by MBP-tagged Stim1 fragments. (n = 3) **(C&D)** Representative Fura-2 profiles showing real-time change in average [Ca^2+^]_i_ in response to 1μM TG in *Stim1-/-* MEFs expressing WT (black) and W430Y mutant Stim1 (red) **(C)** or WT (black) and W430A mutant Stim1 (blue) **(D)**. Gray lines show the empty vector (EV) control in each panel. (n = 3; shown here are average traces from n = 1 with ~20 to 30 cells per group). **(E&F)** Average SOCE **(E)**, and rate of calcium influx after replenishing [Ca^2+^]_e_ **(F)**, calculated from 3 independent experiments in panel **(C&D)**. **(G)** Average basal [Ca^2+^]_i_ in resting *Stim1-/-* MEFs expressing WT (black), W430Y Stim1 (red) or W430A Stim1 (blue). (n = 1 with ~20 to 30 cells per group). **(H)** Mean fluorescence intensity (MFI) of *Stim1-/-* MEFs expressing WT (black), W430Y Stim1 (red) or W430A Stim1 (blue). (n = 3, with 20–30 cells/ group). **(I)** TIRF images of resting and store-depleted HEK 293 cells, co-expressing WT, W430Y or W430A YFP-Stim1 and Orai1-CFP. Scale bar 10 μm. (n > 10 cells).

We have previously shown that α-SNAP binds the SOAR/CAD domain of Stim1 [20]. Given the ability of SOAR/ CAD to bind Orai1 as well as α-SNAP, we sought to determine whether Orai1 and α-SNAP bind distinct or overlapping regions within SOAR/ CAD. SOAR/ CAD exists as a mixture of dimer and tetramer in solution and consists of two long α-helices and an intermediate, relatively less well-defined, region [11, 12, 17]. We expressed two long helical regions of SOAR/ CAD, Stim1 342–382 (CC2 domain) and Stim1 408–442 (CC3 domain) [17] (S1 Fig), tagged with maltose binding protein (MBP) and performed *in vitro* pulldown assays with purified human α-SNAP. α-SNAP bound MBP-Stim1 408–442 but not MBP-Stim1 342–382 (Fig 1A), the domain that has been shown to directly interact with Orai1 272–292 [16]. Hence α-SNAP and Orai1 bind non-overlapping coiled-coil regions of SOAR/ CAD.

To further narrow the interaction between Stim1 408–442 and α-SNAP, we used site-directed mutagenesis and generated several point mutations in MBP-Stim1 408–442 (S2A–S2D Fig). Because Stim, SNAP and Orai were originally identified from genome-wide RNAi screens in *Drosophila* S2 cells [2, 20], we limited our analysis to residues that are conserved across species. To determine whether reduced binding of Stim1 mutants to α-SNAP was associated with inability to activate SOCE, we expressed the respective full length YFP tagged-Stim1 mutants in mouse embryonic fibroblasts (MEF) derived from *Stim1-/-* mice and measured their ability to restore SOCE. In parallel, we analyzed their intracellular localization in resting and store depleted cells and the mutants that failed to show normal distribution were not analyzed further. In general, we found that mutating charged and polar residues did not disrupt binding of SOAR/ CAD to α-SNAP (S2A–S2C Fig). However, mutation of W430 strongly reduced binding between MBP-Stim1 408–442 and α-SNAP (Fig 1B) and (S2D Fig). Furthermore, expression of Stim1 W430Y and W430A mutants in *Stim1-/-* MEFs failed to fully reconstitute thapsigargin (TG) induced SOCE (Fig 1C–1F), while demonstrating normal resting [Ca]_i_ (Fig 1G) and comparable protein expression levels per cell (Fig 1H). Interestingly, while W430Y showed normal subcellular distribution and retained its ability to oligomerize as well as co-cluster Orai1 post store depletion, W430A formed clusters constitutively, and co-clustered Orai1 only partially suggesting additional likely roles in imparting structural stability for this residue (Fig 1I). Taken together, these data demonstrate that α-SNAP binds to Stim1-CC3, a region that is crucial for SOCE but does not overlap with the Orai1 interacting region in the SOAR/ CAD domain of Stim1.

### α-SNAP binds to the hinge region near the Orai1 C-terminus

**Fig 2:**
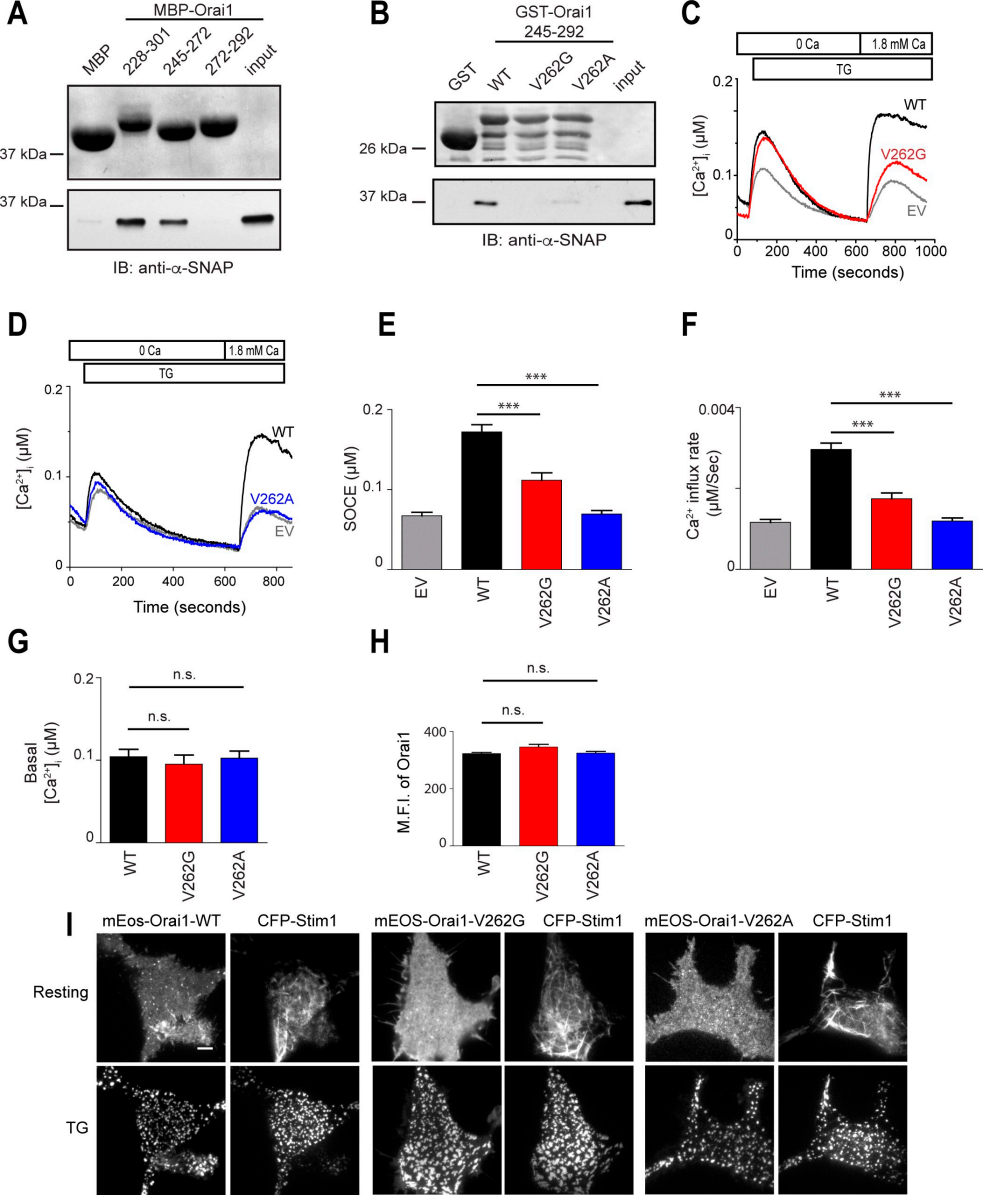
α-SNAP binds to the hinge region near the Orai1 C-terminus. **(A)** *In vitro* binding of α-SNAP to MBP-tagged Orai1 C-terminus 228–301 and its subdomains, Orai1 245–272 and Orai1 272–292. (Top) Ponceau S staining showing the input of MBP-tagged fragments. (Bottom) Immunoblot (IB) for α-SNAP showing α-SNAP protein pull down by MBP-tagged fragments of Orai1. (n = 3) **(B)** *In vitro* binding of α-SNAP to GST-tagged WT, V262G and V262A mutants of Orai1 245–292. (Top) Ponceau S staining showing the GST-tagged protein input. (Bottom) IB for α-SNAP showing α-SNAP protein pull down by GST-tagged Orai1 fragments. (n = 3) **(C&D)** Representative Fura-2 profiles showing real-time change in average [Ca^2+^]_i_ in *Orai1-/-* MEFs expressing WT (black) and V262G mutant Orai1 (red) **(C)** or WT (black) and V262A mutant Orai1 (blue) **(D)**, in response to 1μM TG. Gray lines show the empty vector (EV) control in each panel. (n = 3; shown here are average traces from n = 1 with ~20 to 30 cells per group). **(E&F)** Average SOCE **(E)**, and rate of calcium influx after replenishing [Ca^2+^]_e_ **(F)**, calculated from 3 independent experiments in panel **(C&D)**. **(G)** Average basal [Ca^2+^]_i_ in resting *Orai1-/-* MEFs expressing WT (black), V262G Orai1 (red) or V262A Orai1 (blue). (n = 1 with ~20 to 30 cells per group). **(H)** Mean fluorescence intensity (MFI) of *Orai1-/-* MEFs expressing WT (black), V262G (red) or V262A Orai1 (blue). (n = 3, with 20–30 cells per group). **(I)** TIRF images of resting and store-depleted HEK 293 cells, co-expressing WT or V262 mutant mEOS3.2-Orai1 and CFP-Stim1. Scale bar 10 μm. (n > 10 cells).

We have previously shown that α-SNAP directly binds the C-terminus of Orai1 [20]. However, given that SOAR/ CAD also binds the Orai1 C-terminus [16], we next sought to map the α-SNAP interacting region in Orai1 C-terminus. We constructed MBP fusion proteins of the entire C-terminus of Orai1, including the transmembrane region 4 (TM4) or its helical regions; Orai1 245–272 and Orai1 272–292 (S1 Fig) and assessed the ability of these respective fusion proteins to bind purified α-SNAP in *in-vitro* pull down assays (Fig 2A). Interestingly, α-SNAP bound MBP-Orai1 245–272, but not MBP-Orai1 272–292, the ~20 amino acid long coiled-coil region that binds SOAR/ CAD [16]. These data demonstrate that similar to the case of SOAR/ CAD, non-overlapping regions of Orai1 C-terminus are involved in associating with Stim1 and α-SNAP (Fig 2A).

Next, we used site-directed mutagenesis and found that mutating four residues, 262–265 (VHSK) to Alanine in the Orai1 C-terminus hinge region [26] showed significantly reduced binding to α-SNAP (S3A Fig). Analysis of individual residues within and outside the hinge (S3B and S3C Fig) revealed V262 as a crucial residue (Fig 2B). Remarkably, expression of full-length mEOS3.2 tagged-Orai1 V262G or V262A mutant in *Orai1-/-* MEFs failed to fully restore SOCE (Fig 2C–2F) but did not show constitutive calcium influx (Fig 2G) or defects in the cell surface localization of Orai1 (Fig 2H). V262 mutants also retained the ability to co-cluster with Stim1 in store-depleted cells (Fig 2I). Taken together, these data indicate that direct binding of α-SNAP within the Orai1 C-terminus hinge enables SOCE *via* Orai1 channels.

### α-SNAP binds a membrane proximal region of the Orai1 N-terminus

**Fig 3:**
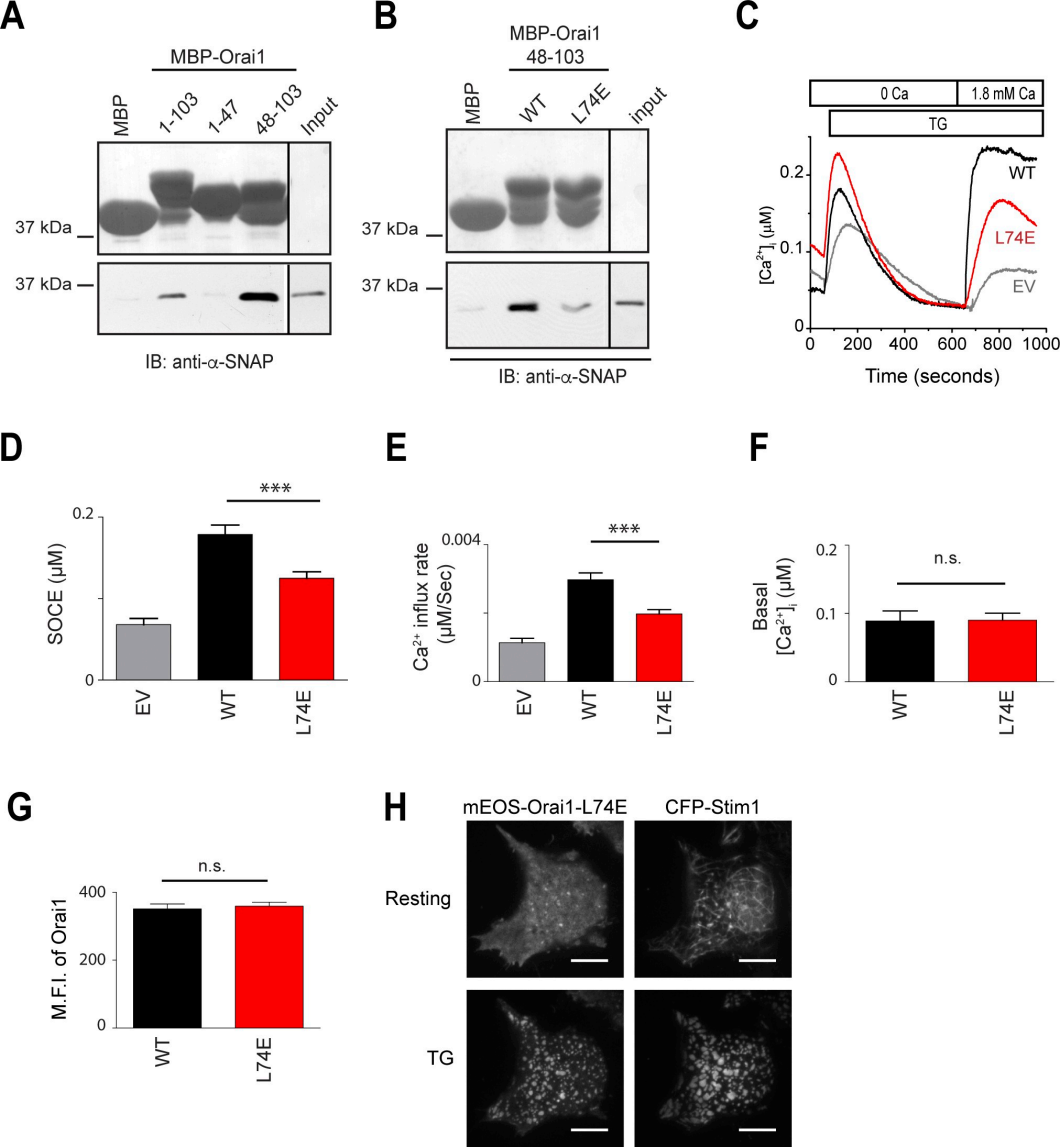
α-SNAP binds a membrane proximal region of Orai1 N-terminus. **(A)** *In vitro* binding of α-SNAP to MBP-tagged Orai1 N-terminus 1–103 and its subdomains, Orai1 1–47 and Orai1 48–103. (Top) Ponceau S staining showing the input of MBP-tagged Orai1-N terminus fragments. (Bottom) IB for α-SNAP showing α-SNAP protein pull down by MBP-tagged fragments of Orai1-N terminus. (n = 3) **(B)** *In vitro* binding of α-SNAP to MBP-tagged WT and L74E Orai1 48–103. (Top) Ponceau S staining showing the MBP-tagged protein input. (Bottom) IB for α-SNAP showing α-SNAP protein pull down by MBP-tagged WT and L74E Orai1 48–103. (n = 3) **(C)** Representative Fura-2 profiles showing real-time change in average [Ca^2+^]_i_ in *Orai1-/-* MEFs expressing WT (black) or L74E mutant Orai1 (red), in response to 1μM TG. Gray line shows the EV control. (n = 3; shown here are average traces from n = 1 with ~20 to 30 cells per group). **(D&E)** Average SOCE **(D)**, and rate of calcium influx after replenishing [Ca^2+^]_e_ **(E)**, calculated from 3 independent experiments in panel **(C)**. **(F)** Average basal [Ca^2+^]_i_ in resting *Orai1-/-* MEFs expressing WT (black) or L74E Orai1 (red). (n = 1 with ~20 to 30 cells per group). **(G)** Mean fluorescence intensity (MFI) of *Orai1-/-* MEFs expressing WT (black) or L74E Orai1 (red). (n = 3, with 20–30 cells per group/ experiment). **(H)** TIRF images of resting and store-depleted HEK 293 cells, co-expressing mEOS3.2-Orai1 L74E and CFP-Stim1. Scale bar 10 μm. (n>10).

Previously, we have observed a faint association between GST-tagged Orai1 N-terminus and purified α-SNAP [20]. Therefore, we first confirmed the interaction between Orai1 N-terminus and α-SNAP by generating MBP-tagged Orai1 1–103 and its two fragments based on previous domain analysis [11, 12, 19, 20, 27] (S1 Fig). We found that α-SNAP bound much more strongly to MBP-Orai1 48–103 when compared to Orai1 1–103, but not to MBP-Orai1 1–47 (Fig 3A).

We next sought to identify the residues crucial for binding between α-SNAP and the Orai1 N-terminus. Mutations of several charged residues in Orai1 N-terminus did not reduce binding to α-SNAP. However, mutation of L74 resulted in significantly reduced binding (Fig 3B). L74, residing in the Extended Transmembrane Orai1 N-Terminal (ETON) region, has been shown to be crucial for SOCE as well as coupling to Orai activating Stim fragment (OASF) [28, 29]. In accordance with previous studies, Orai1 L74E failed to fully restore SOCE when expressed in *Orai1-/-* MEFs (Fig 3C–3E). Resting Orai1 L74E expressing cells showed normal basal [Ca^2+^]_i_ (Fig 3F) and cell surface expression (Fig 3G). Upon store-depletion, the ability of Orai1 L74E to co-cluster with full length Stim1 remained unchanged (Fig 3H). These data demonstrate that L74 in the Orai1 N-terminus is involved in the functional interaction with α-SNAP.

### α-SNAP binds Stim1 as well as Orai1 with high affinity

**Fig 4:**
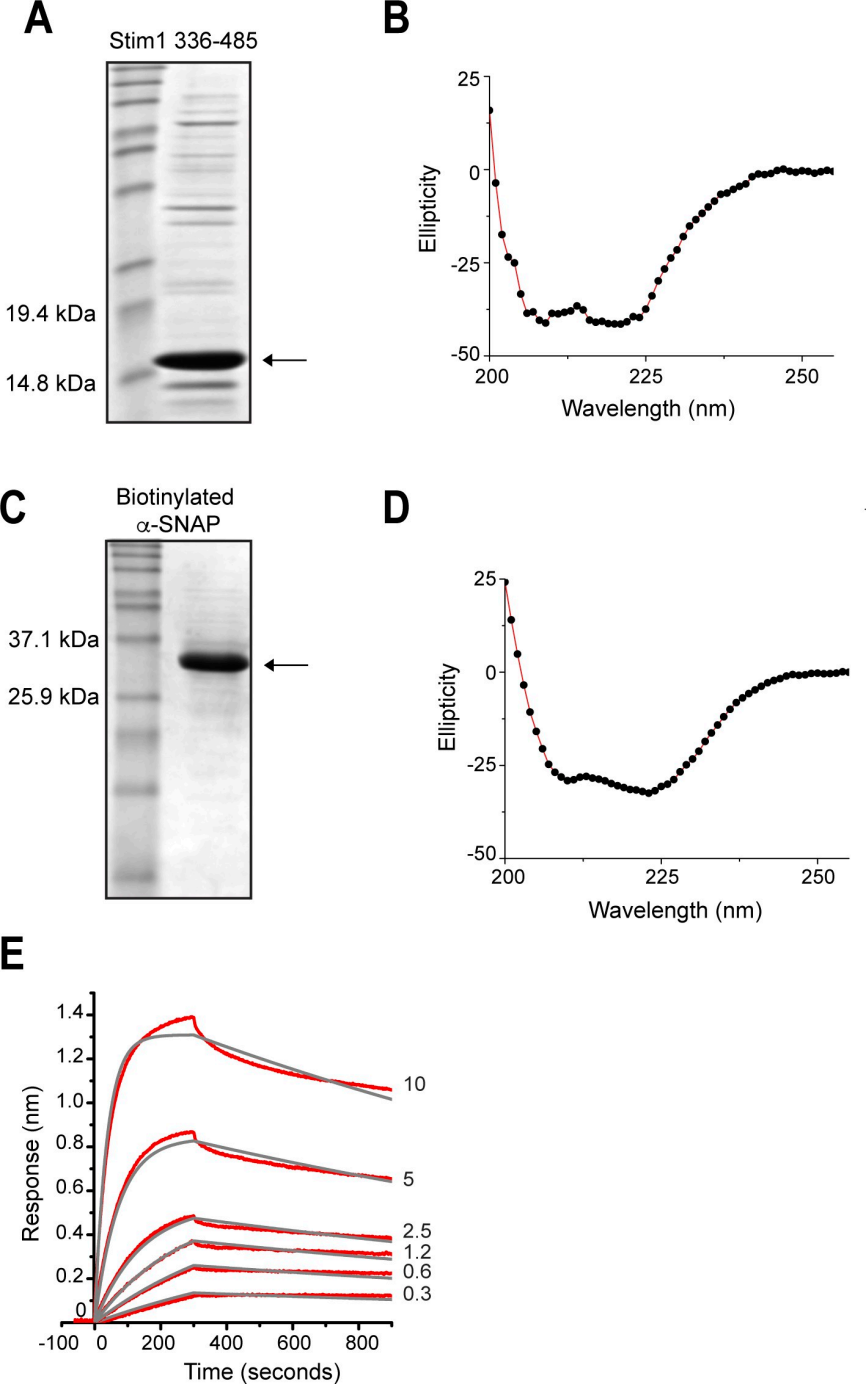
α-SNAP binds Stim1 with high affinity. **(A)** SDS-PAGE showing purified human Stim1 336–485. **(B)** CD spectra of purified Stim1 336–485. **(C)** SDS-PAGE showing Avi-Tagged human α-SNAP expressed, purified and biotinylated using BirA ligase. **(D)** CD spectra of purified and biotinylated human α-SNAP. **(E)** BLI assay to measure the binding parameters of increasing concentrations of soluble Stim1 336–485 (0.3 to 10 μM) to biotinylated α-SNAP immobilized on streptavidin biosensor (ForteBio Inc., USA). Raw data (red lines) were globally fit to 1:1 binding model (gray lines) using the ForteBio Inc. software. (n>2).

Our previous *in vitro* pull down studies have suggested that α-SNAP binds to GST-tagged CAD domain of Stim1 with relatively higher affinity compared to GST-tagged Orai1-C-terminus and N-terminus [20]. To confirm these observations, and measure the binding parameters and affinity of α-SNAP for Stim and Orai we used Bio-layer interferometry (BLI) (ForteBio Inc., USA). BLI allows measurement of protein-protein interactions without the need for a tag. We expressed and purified Stim1 336–485 (S1 Fig), as our attempts to purify CAD/SOAR were unsuccessful [11] (Fig 4A). The circular dichroism (CD) spectra of purified Stim1 336–485 showed 44% alpha helical conformation (Fig 4B) [11]. In parallel, we expressed C-terminal Avi-Tagged human α-SNAP, biotinylated using BirA ligase and purified it (Fig 4C). CD spectra of purified α-SNAP showed the expected 73% alpha helical conformation (Fig 4D) [24]. We captured biotinylated α-SNAP on the streptavidin biosensor (ForteBio Inc., USA) and tested its binding to soluble Stim1 336–485 at increasing concentrations. The raw data were globally fit to 1:1 binding model using the ForteBio Inc. software and the dissociation constant (K_D_) was found to be 158±1.2 nM (Fig 4E) and (Table 1).

**Table 1:** Binding parameters for α-SNAP and Stim1 336–485.

*K*_*D*_ (nM)	*k*_*on*_ (M^-1^s^-1^)	*k*_*off*_ (s^-1^)	R^2^
158 ± 1.2	2.68 x 10^3^ ± 12.5	4.22 x 10^−4^ ± 2.55 x 10^−6^	0.997

The experimental data in Fig 4E were fit to the single-site model of ligand binding (1:1 binding model) using ForteBIO data analysis software to obtain association rate constant (*k*_*on*_), dissociation rate constant (*k*_*off*_), and dissociation constant (*K*_*D*_) values.

**Fig 5:**
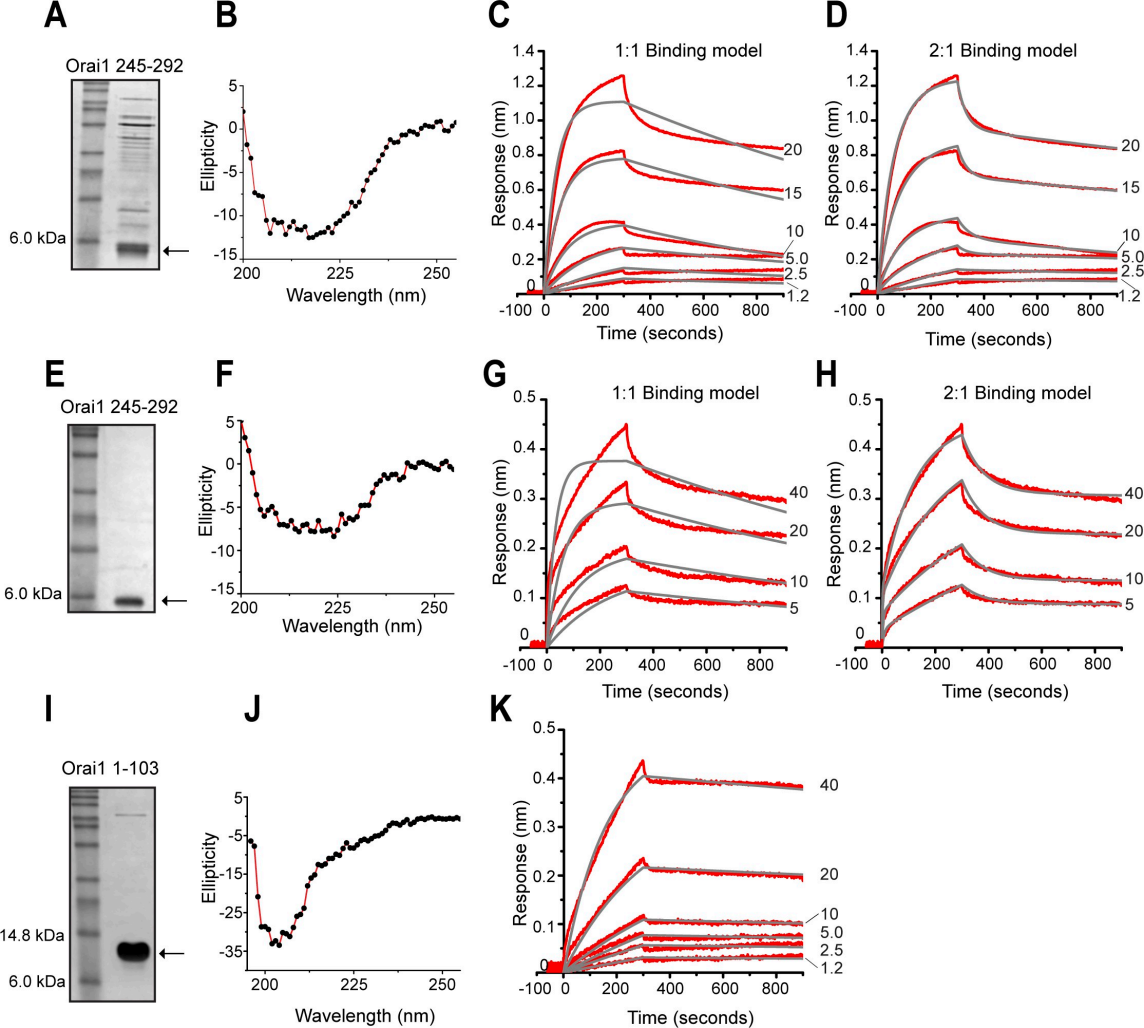
α-SNAP harbors comparable affinity for Orai1 and Stim1. **(A&E)** SDS-PAGE showing purified human Orai1 245–292 **(A)** and Orai1 245–292 peptide **(E)**. **(B&F)** CD spectra of purified untagged Orai1 245–292 **(B)** and Orai1 245–292 peptide **(F)**. **(C,D & G,H)** BLI assays to measure the binding parameters of increasing concentrations of purified Orai1 245–292 (1.2 to 20 μM) **(C,D)** or Orai1 245–292 peptide (5 to 40 μM) **(G,H)** to biotinylated α-SNAP immobilized on the streptavidin biosensor (Forte Bio Inc.). Raw data (red lines) were globally fit to 1:1 **(C&G)** and 2:1 **(D&H)** binding models (gray lines). (n>2 each). **(I)** SDS-PAGE showing purified human Orai1 1–103. **(J)** CD spectra of purified untagged Orai1 1–103. **(K)** BLI assay to measure the binding parameters of increasing concentrations of soluble Orai1 1–103 (1.2 to 40 microM) to biotinylated α-SNAP immobilized on the streptavidin biosensor (ForteBio Inc.). Raw data (red lines) were globally fit to 1:1 binding model (gray lines). (n>2).

We next expressed and purified untagged Orai1 C-terminal 245–292 (S1 Fig) and (Fig 5A), confirmed its folding using CD spectra (Fig 5B), which showed 50% alpha helical content, and measured the kinetics of its binding to α-SNAP using BLI. As described in Fig 4, we captured biotinylated α-SNAP on the biosensor and incubated with varying concentrations of Orai1 245–292. The data obtained were globally fit to 1:1 (Fig 5C) and 2:1 (Fig 5D) binding models and the K_D_ obtained from the 2:1 binding model, which yielded a better fit, was 314±1.86 nM (Table 2). The 2:1 model assumes that the analyte, Orai1 245–292, bound to two independent sites on the ligand i.e. α-SNAP. Because impurities or aggregates in purified proteins can also sometimes give bimodal binding, we verified these data using a commercially synthesized Orai1 245–292 peptide. The CD spectra, binding parameters and affinity of the synthesized Orai1 245–292 peptide were overall similar to purified Orai1 245–292 (Fig 5E–5H) and the K_D_ obtained from the 2:1 binding model was 198±19.5 nM (Table 3).

**Table 2:** Binding parameters for α-SNAP and purified Orai1 245–292.

**1:1 binding model**			
***K***_***D***_ **(nM)**	***k***_***on***_ **(M**^**-1**^**s**^**-1**^**)**	***k***_***off***_ **(s**^**-1**^**)**	**R** ^**2**^
548 ± 7.2	1.08 x 10^3^ ± 9.86	5.95 x 10^−4^ ± 5.57 x 10^−6^	0.989
**2:1 binding model**			
***K***_***D1***_ **(nM)**	***k***_***on1***_ **(M**^**-1**^**s**^**-1**^**)**	***k***_***off1***_ **(s**^**-1**^**)**	**R** ^**2**^
314 ± 1.9	6.78 x 10^2^ ± 6.03	2.13 x 10^−4^ ± 4.02 x 10^−6^	0.998
***K***_***D2***_ **(μM)**	***k***_***on2***_ **(M**^**-1**^**s**^**-1**^**)**	***k***_***off2***_ **(s**^**-1**^**)**	
202 ± 71.9	1.52 x 10^2^ ± 53.9	3.06 x 10^−2^ ± 5.92 x 10^−4^	

The experimental data in Fig 5C and 5D were fit to the single-site model (1:1 binding model) and the two-site model (2:1 binding model) of ligand binding, respectively. The association rate constant (*k*_*on*_), dissociation rate constant (*k*_*off*_), and dissociation constant (*K*_*D*_) values were obtained using the ForteBIO data analysis software.

**Table 3:** Binding parameters for α-SNAP and Orai1 245–292 peptide.

**1:1 binding model**			
***K***_***D***_ **(nM)**	***k***_***on***_ **(M**^**-1**^**s**^**-1**^**)**	***k***_***off***_ **(s**^**-1**^**)**	**R** ^**2**^
369 ± 11.9	1.37 x 10^3^ ± 32.7	5.06 x 10^−4^ ± 1.1 x 10^−5^	0.953
**2:1 binding model**			
***K***_***D1***_ **(nM)**	***k***_***on1***_ **(M**^**-1**^**s**^**-1**^**)**	***k***_***off1***_ **(s**^**-1**^**)**	**R** ^**2**^
198 ± 19.5	2.84 x 10^2^ ± 3.17	5.62 x 10^−5^ ± 5.5 x 10^−6^	0.996
***K***_***D2***_ **(nM)**	***k***_***on2***_ **(M**^**-1**^**s**^**-1**^**)**	***k***_***off2***_ **(s**^**-1**^**)**	
715 ± 54.1	3.90 x 10^4^ ± 2.87 x 10^3^	2.79 x 10^−2^ ± 5.06 x 10^−4^	

The experimental data in Fig 5G and 5H were fit to the single-site model (1:1 binding model) and the two-site model (2:1 binding model) of ligand binding, respectively. The association rate constant (*k*_*on*_), dissociation rate constant (*k*_*off*_), and dissociation constant (*K*_*D*_) values were obtained using the ForteBIO data analysis software.

To determine the affinity of interaction between α-SNAP and the Orai1 N-terminus, we expressed and purified a large untagged Orai1 1–103 fragment (S1 Fig) and (Fig 5I), as we were unable to purify shorter regions. Orai1 1–103 purified relatively easily, showed minimal degradation and the CD spectra showed 53% alpha-helical content (Fig 5J). We measured the binding parameters of α-SNAP to increasing concentrations of Orai1 1–103 using BLI and fitted the data using 1:1 binding model (Fig 5K). The K_D_ obtained from these data was 763±20.7 nM (Table 4). Taken together, these data suggest that the affinities of α-SNAP for Orai1 245–292 and Stim1 336–485 are comparable. Although, the association of α-SNAP with Orai1 1–103 was relatively weaker, the affinity is still higher in comparison to what has been previously reported for Orai1 N-terminal fragments and OASF interaction [29].

**Table 4:** Binding parameters for α-SNAP and Orai1 1–103.

*K*_*D*_ (nM)	*k*_*on*_ (M^-1^s^-1^)	*k*_*off*_ (s^-1^)	R^2^
763 ± 20.7	1.54 x 10^2^ ± 1.21	1.17 x 10^−4^ ± 3.04 x 10^−6^	0.997

The experimental data in Fig 5K were fit to the single-site model of ligand binding (1:1 binding model) and association rate constant (*k*_*on*_), dissociation rate constant (*k*_*off*_), and dissociation constant (*K*_*D*_) values were obtained using the ForteBIO data analysis software.

## Discussion

In this study, we have identified the minimal sub-domains of Orai1 and Stim1 involved in associating with α-SNAP, a calcium release activated calcium (CRAC) channel component necessary for the functional assembly and selectivity of Orai1 multimers. By mutating specific residues within these domains, our data further suggest that failure to bind α-SNAP results in defective SOCE despite robust co-clustering of Stim, Orai in the endoplasmic reticulum-plasma membrane (ER-PM) junctions. These observations reinforce our previous findings [14, 20] that the step of Orai Stim co-clustering within ER-PM junctions can be dissociated from SOCE at a molecular level.

Previous studies have implicated the SOAR/CAD subdomain, Stim1-CC3, in Stim oligomerization and W430 falls in the putative dimer interface region [17]. It was therefore surprising to see normal oligomerization of Stim1 W430Y and co-clustering with Orai1, post store-depletion. One possibility is that the substitution of tryptophan 430 to tyrosine allowed the formation of hydrogen bonds between full length Stim proteins in the W430Y expressing cells. However, the amplitude of SOCE was still significantly reduced suggesting additional functions for this domain. On the other hand, constitutive oligomerization of Stim1-W430A and its failure to efficiently co-cluster Orai1 or stimulate SOCE could suggest potential structural defects introduced by this mutation, which may preclude functional association with itself as well as α-SNAP. Collectively, these data point to additional roles for Stim1 CC3 that are beyond Stim1 oligomerization and facilitated by high affinity contacts with α-SNAP.

Previously, multiple mutations introduced in the Orai1 C-terminus hinge have revealed the importance of this region in Orai1 gating by inducing hypothetical, allosteric changes in the neighboring Orai1-transmembrane (TM) regions and the Orai1-N-terminus to activate constitutive calcium influx [30]. However, unlike the earlier reported mutations of Orai1 in this region [30], V262G or V262A mutations neither resulted in constitutively active nor non-selective Orai1 by themselves, suggesting that the structure of Orai1 C-terminal hinge region (261–265) per se was not altered in V262 mutants. The hinge region precedes the helix that directly binds Stim1 CC2 and therefore it is also unlikely that the structure of Stim1-CC2 helix was disrupted. Therefore, the simplest possible explanation in view of our data and earlier reports is that reduced SOCE in V262 mutants results from defective functional interaction with α-SNAP. In light of high affinity of α-SNAP for Orai1 245–292, its ability to bind Orai1 C- and N-termini independently of Stim1 and our previous findings [14], these data suggest that the binding of α-SNAP to Orai1 facilitates structural shifts within Orai1 which enable SOCE.

The structure of Sec17, the yeast homolog of α-SNAP, consists of 14 α helices with the first nine forming a twisted sheet and the last 5 forming a globular bundle [31]. Previous studies regarding the association of α-SNAP with SNAREs have suggested that SNAPs recognize general surface features of interacting proteins rather than individual residues. Therefore, while we have identified specific residues within Orai1 and Stim1 that are crucial for *in vitro* binding, these data do not rule out the involvement of additional residues within the overall interacting region despite apparently normal *in vitro* binding of additional mutants. Likewise, although the residues of Stim1 and Orai1 that we have identified here are crucial for association with α-SNAP, the data presented here do not rule out additional roles for them, for instance, in imparting the overall structural stability and/or allosteric effects on associations between Stim1 and Orai1. While any mutation always holds the possibility that it may affect Orai, Stim structure and interactions, the robust co-clustering that we observed in several of our mutants argues that these mutations do not affect Orai-Stim physical interaction, while affecting SOCE, further indicating that α-SNAP facilitates their functional interaction.

It is surprising that the affinity of α-SNAP for Orai1 as well as Stim1 was significantly higher than the previously reported affinity of Stim1 for Orai1 [29]. A major goal of affinity measurements in this study was to potentially determine the sequence of recruitment of these three key components to the CRAC channel supramolecular complex. However, given that the affinities of α-SNAP for Stim1 336–485 and Orai1 C-terminus are not very different, the sequence of recruitment could not be discerned from these studies. Furthermore, previous studies have suggested that Stim1-CC3, as well as the Orai1 interacting CC2 domains, could be masked in resting cells by CC1 domain of Stim1 [17]. Similar allosteric constraints have been reported for binding of CAD/ SOAR to membrane proximal regions of the Orai1 N-terminus. Therefore, accessibility of sub-domains involved in the intermolecular interactions will also be a crucial determinant of the sequence of recruitment of these proteins *in vivo*.

In summary, we have identified novel high affinity intermolecular interactions necessary for the activation of SOCE *via* Orai1. The interaction interfaces identified here could, in future, be useful for designing peptide or small molecule inhibitors of SOCE.

## Supporting information

S1 Fig Schematics of Stim1 and Orai1 domains analyzed in this paper. (TIF)

S2 Fig *In vitro* binding of α-SNAP to MBP-tagged mutants of Stim1 408–442. **(A)** Stim1 408–442 basic residue mutants. **(B)** Stim1 408–442 acidic residue mutants. **(C)** Stim1 408–442 polar residue mutants. **(D)** Stim1 408–442 hydrophobic residue mutants. (Top) Ponceau S staining showing the input of MBP-tagged Stim1 408–442 mutants. (Bottom) Western Blot for α-SNAP showing α-SNAP pull down by various mutants.
(TIF)

S3 Fig *In vitro* binding of α-SNAP to GST-tagged mutants of Orai1 245–292. (**A-C)** (Top) Ponceau S staining showing the input of GST-tagged Orai1 245–292 mutants. (Bottom) Western Blot for α-SNAP showing α-SNAP pull down by respective mutants.
(TIF)

## References

[1] Vig M. and Kinet J.P., The long and arduous road to CRAC. Cell Calcium, 2007. 42(2): p. 157–62. doi: 10.1016/j.ceca.2007.03.008 17517435 PMC2001162

[2] Vig M., et al., CRACM1 is a plasma membrane protein essential for store-operated Ca2+ entry. Science, 2006. 312(5777): p. 1220–3. doi: 10.1126/science.1127883 16645049 PMC5685805

[3] Zhang S.L., et al., Genome-wide RNAi screen of Ca(2+) influx identifies genes that regulate Ca(2+) release-activated Ca(2+) channel activity. Proc Natl Acad Sci U S A, 2006. 103(24): p. 9357–62. doi: 10.1073/pnas.0603161103 16751269 PMC1482614

[4] Zhang S.L., et al., STIM1 is a Ca2+ sensor that activates CRAC channels and migrates from the Ca2+ store to the plasma membrane. Nature, 2005. 437(7060): p. 902–5. doi: 10.1038/nature04147 16208375 PMC1618826

[5] Feske S., et al., A mutation in Orai1 causes immune deficiency by abrogating CRAC channel function. Nature, 2006. 441(7090): p. 179–85. doi: 10.1038/nature04702 16582901

[6] Liou J., et al., STIM is a Ca2+ sensor essential for Ca2+-store-depletion-triggered Ca2+ influx. Curr Biol, 2005. 15(13): p. 1235–41. doi: 10.1016/j.cub.2005.05.055 16005298 PMC3186072

[7] Liou J., et al., Live-cell imaging reveals sequential oligomerization and local plasma membrane targeting of stromal interaction molecule 1 after Ca2+ store depletion. Proc Natl Acad Sci U S A, 2007. 104(22): p. 9301–6. doi: 10.1073/pnas.0702866104 17517596 PMC1890489

[8] Soboloff J., et al., STIM proteins: dynamic calcium signal transducers. Nature reviews. Molecular cell biology, 2012. 13(9): p. 549–65. doi: 10.1038/nrm3414 22914293 PMC3458427

[9] Luik R.M., et al., Oligomerization of STIM1 couples ER calcium depletion to CRAC channel activation. Nature, 2008. 454(7203): p. 538–42. doi: 10.1038/nature07065 18596693 PMC2712442

[10] Prakriya M. and Lewis R.S., Store-Operated Calcium Channels. Physiol Rev, 2015. 95(4): p. 1383–436. doi: 10.1152/physrev.00020.2014 26400989 PMC4600950

[11] Yuan J.P., et al., SOAR and the polybasic STIM1 domains gate and regulate Orai channels. Nature cell biology, 2009. 11(3): p. 337–43. doi: 10.1038/ncb1842 19182790 PMC2663385

[12] Park C.Y., et al., STIM1 clusters and activates CRAC channels via direct binding of a cytosolic domain to Orai1. Cell, 2009. 136(5): p. 876–90. doi: 10.1016/j.cell.2009.02.014 19249086 PMC2670439

[13] Wu M.M., Covington E.D., and Lewis R.S., Single-molecule analysis of diffusion and trapping of STIM1 and Orai1 at endoplasmic reticulum-plasma membrane junctions. Mol Biol Cell, 2014. 25(22): p. 3672–85. doi: 10.1091/mbc.E14-06-1107 25057023 PMC4230625

[14] Li P., et al., alpha-SNAP regulates dynamic, on-site assembly and calcium selectivity of Orai1 channels. Mol Biol Cell, 2016. 27(16): p. 2542–53. doi: 10.1091/mbc.E16-03-0163 27335124 PMC4985256

[15] Calloway N., et al., Molecular clustering of STIM1 with Orai1/CRACM1 at the plasma membrane depends dynamically on depletion of Ca2+ stores and on electrostatic interactions. Molecular biology of the cell, 2009. 20(1): p. 389–99. doi: 10.1091/mbc.e07-11-1132 18987344 PMC2613096

[16] Stathopulos P.B., et al., STIM1/Orai1 coiled-coil interplay in the regulation of store-operated calcium entry. Nat Commun, 2013. 4: p. 2963. doi: 10.1038/ncomms3963 24351972 PMC3927877

[17] Yang X., et al., Structural and mechanistic insights into the activation of Stromal interaction molecule 1 (STIM1). Proceedings of the National Academy of Sciences of the United States of America, 2012. 109(15): p. 5657–62. doi: 10.1073/pnas.1118947109 22451904 PMC3326449

[18] Zhou Y., et al., STIM1 gates the store-operated calcium channel ORAI1 in vitro. Nature structural & molecular biology, 2010. 17(1): p. 112–6. doi: 10.1038/nsmb.1724 20037597 PMC2902271

[19] Palty R. and Isacoff E.Y., Cooperative Binding of Stromal Interaction Molecule 1 (STIM1) to the N and C Termini of Calcium Release-activated Calcium Modulator 1 (Orai1). J Biol Chem, 2016. 291(1): p. 334–41. doi: 10.1074/jbc.M115.685289 26546674 PMC4697168

[20] Miao Y., et al., An essential and NSF independent role for alpha-SNAP in store-operated calcium entry. Elife, 2013. 2: p. e00802. doi: 10.7554/eLife.00802 23878724 PMC3713520

[21] Miao Y., et al., Na+ influx via Orai1 inhibits intracellular ATP induced mTORC2 signaling to disrupt CD4 T cell gene expression and differentiation. Elife, 2017. 6. doi: 10.7554/eLife.25155 28492364 PMC5459575

[22] Vig M., et al., Defective mast cell effector functions in mice lacking the CRACM1 pore subunit of store-operated calcium release-activated calcium channels. Nat Immunol, 2008. 9(1): p. 89–96. doi: 10.1038/ni1550 18059270 PMC2377025

[23] Peinelt C., et al., Amplification of CRAC current by STIM1 and CRACM1 (Orai1). Nat Cell Biol, 2006. 8(7): p. 771–3. doi: 10.1038/ncb1435 16733527 PMC5685802

[24] Marz K.E., Lauer J.M., and Hanson P.I., Defining the SNARE complex binding surface of alpha-SNAP: implications for SNARE complex disassembly. J Biol Chem, 2003. 278(29): p. 27000–8. doi: 10.1074/jbc.M302003200 12730228

[25] Whitmore L. and Wallace B.A., Protein secondary structure analyses from circular dichroism spectroscopy: methods and reference databases. Biopolymers, 2008. 89(5): p. 392–400. doi: 10.1002/bip.20853 17896349

[26] Hou X., et al., Crystal structure of the calcium release-activated calcium channel Orai. Science, 2012. 338(6112): p. 1308–13. doi: 10.1126/science.1228757 23180775 PMC3695727

[27] Zheng H., et al., Differential roles of the C and N termini of Orai1 protein in interacting with stromal interaction molecule 1 (STIM1) for Ca2+ release-activated Ca2+ (CRAC) channel activation. J Biol Chem, 2013. 288(16): p. 11263–72. doi: 10.1074/jbc.M113.450254 23447534 PMC3630893

[28] Muik M., et al., A Cytosolic Homomerization and a Modulatory Domain within STIM1 C Terminus Determine Coupling to ORAI1 Channels. The Journal of biological chemistry, 2009. 284(13): p. 8421–6. doi: 10.1074/jbc.C800229200 19189966 PMC2659200

[29] Derler I., et al., The extended transmembrane Orai1 N-terminal (ETON) region combines binding interface and gate for Orai1 activation by STIM1. J Biol Chem, 2013. 288(40): p. 29025–34. doi: 10.1074/jbc.M113.501510 23943619 PMC3790000

[30] Zhou Y., et al., The remote allosteric control of Orai channel gating. PLoS Biol, 2019. 17(8): p. e3000413. doi: 10.1371/journal.pbio.3000413 31469825 PMC6742413

[31] Rice L.M. and Brunger A.T., Crystal structure of the vesicular transport protein Sec17: implications for SNAP function in SNARE complex disassembly. Molecular cell, 1999. 4(1): p. 85–95. doi: 10.1016/s1097-2765(00)80190-2 10445030

