# Supplementary figures and images for: High affinity associations with α-SNAP enable calcium entry via Orai1 channels
Source: PLoS One. 2021 Oct 15;16(10):e0258670. doi: 10.1371/journal.pone.0258670 (PMC8519427; doi:10.1371/journal.pone.0258670)

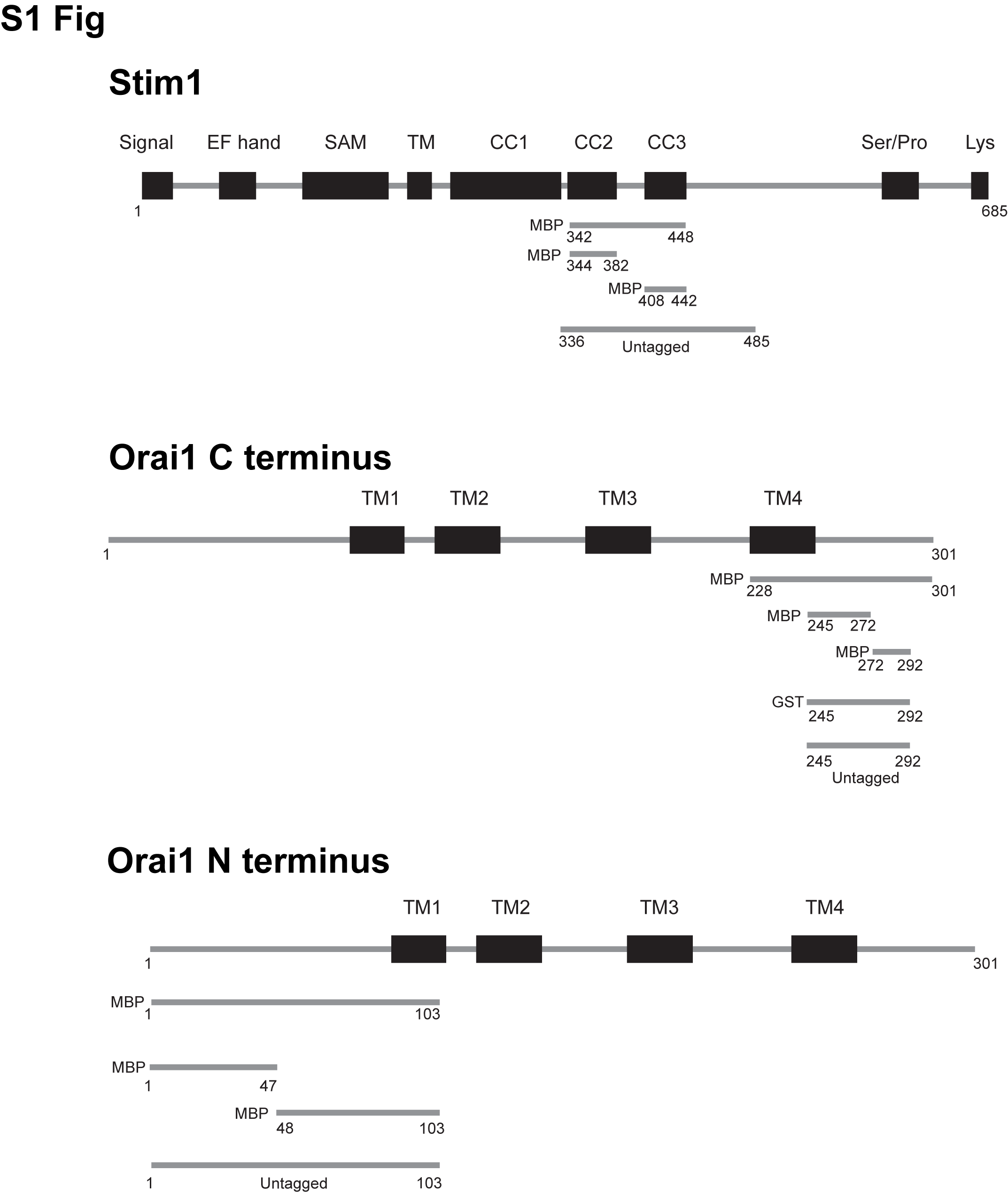

Supplement: S1 Fig — (TIF) [file pone.0258670.s001.tif]

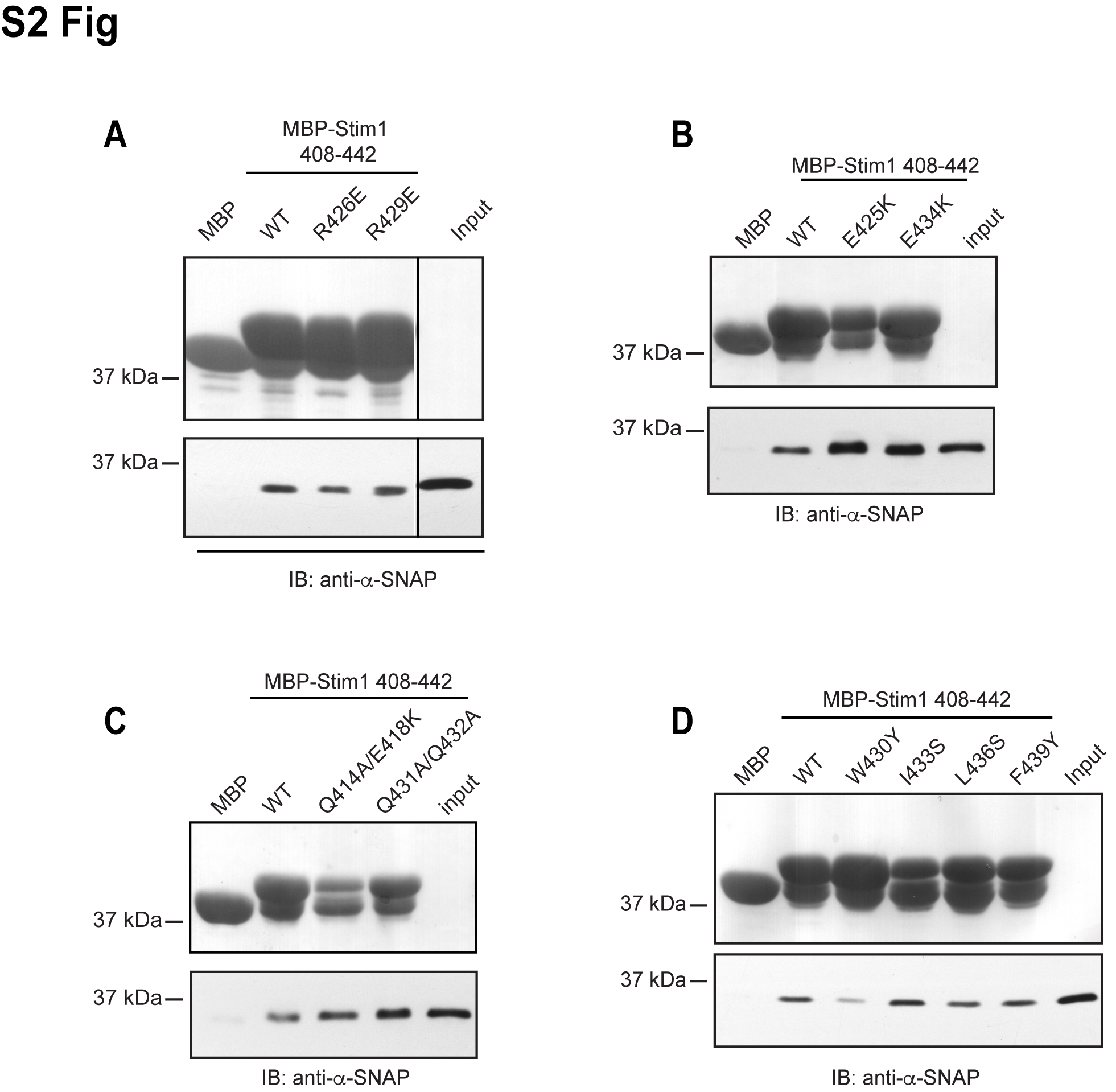

Supplement: S2 Fig — (A) Stim1 408–442 basic residue mutants. (B) Stim1 408–442 acidic residue mutants. (C) Stim1 408–442 polar residue mutants. (D) Stim1 408–442 hydrophobic residue mutants. (Top) Ponceau S staining showing the input of MBP-tagged Stim1 408–442 mutants. (Bottom) Western Blot for α-SNAP showing α-SNAP pull down by various mutants. (TIF) [file pone.0258670.s002.tif]

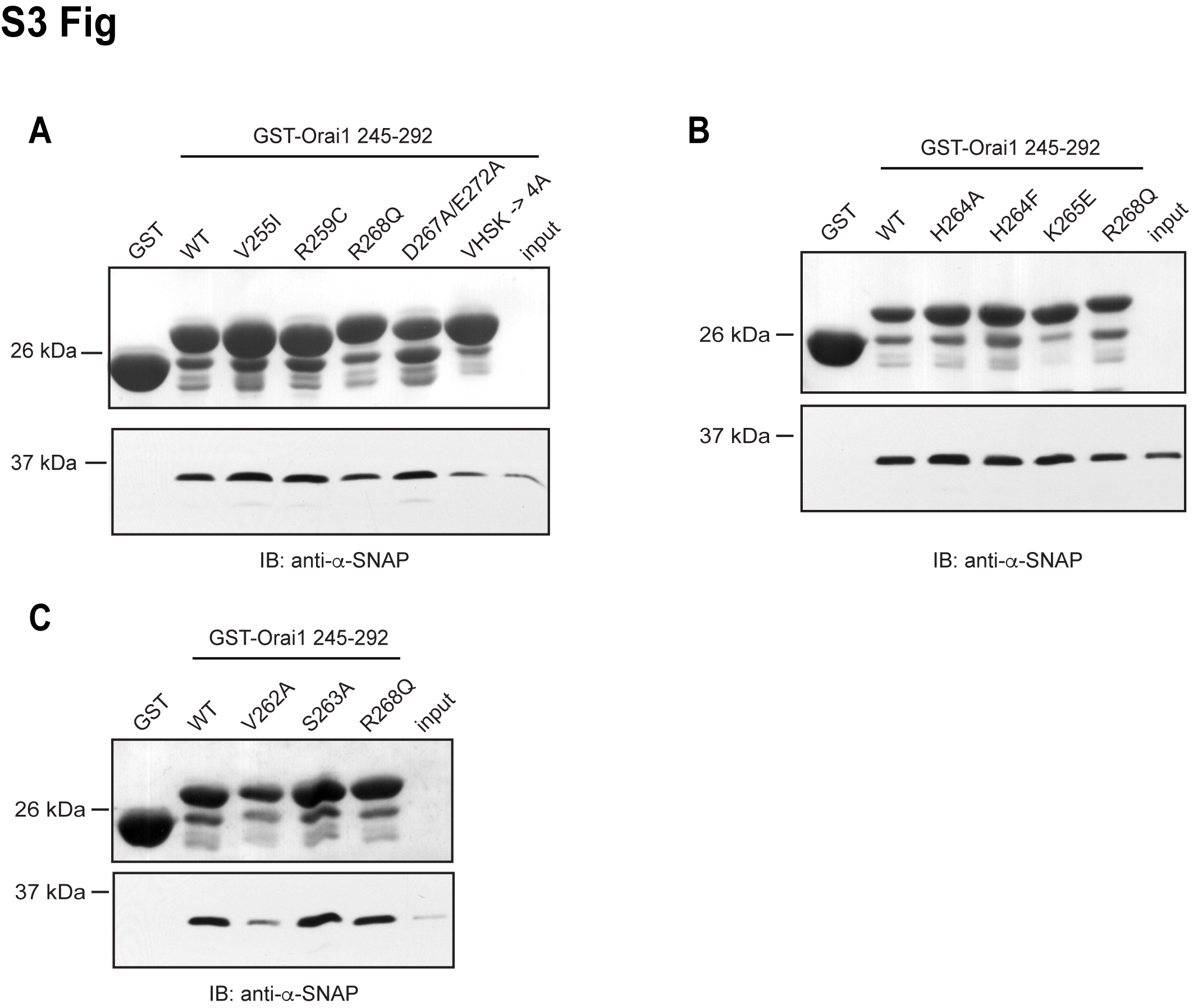

Supplement: S3 Fig — (A-C) (Top) Ponceau S staining showing the input of GST-tagged Orai1 245–292 mutants. (Bottom) Western Blot for α-SNAP showing α-SNAP pull down by respective mutants. (TIF) [file pone.0258670.s003.tif]
